# Citrate polymer optical fiber for measuring refractive index based on LSPR sensor

**DOI:** 10.1038/s41598-024-69083-2

**Published:** 2024-08-11

**Authors:** Fatemeh Arefnia, Mohammad Ismail Zibaii, Azam Layeghi, Soroush Rostami, Mohammad-Mahdi Babakhani-Fard, Fatemeh Mortazavi Moghadam

**Affiliations:** Laser and Plasma Research Institute, Shahid Beheshti University, Tehran, 19839 69411 Iran

**Keywords:** Polymer optical fiber, Local surface plasmon, Refractive index, Poly (octamethylene maleate citrate) (POMC), Poly (octamethylene citrate) (POC), Biosensors, Fibre optics and optical communications, Optical sensors

## Abstract

Fiber optic localized surface plasmon resonance (LSPR) sensors have become an effective tool in refractive index (RI) detection for biomedical applications because of their high sensitivity. However, using conventional optical fiber has caused limitations in implanting the sensor in the body. This research presents the design and construction of a new type of polymer-based LSPR sensors to address this issue. Also, finite element method (FEM) is used to design the sensor and test it theoretically. The proposed polymer optical fiber (POF) based on citrate is biocompatible, flexible, and degradable, with a rate of 22% and 27 over 12 days. The step RI structure utilizes two polymers for light transmission: poly (octamethylene maleate citrate) (POMC) as the core and poly (octamethylene citrate) (POC) as the cladding. The POF core and cladding diameters and lengths are 700 µm, 1400 µm, and 7 cm, respectively. The coupling efficiency of light to the POF was enhanced using a microsphere fiber optic tip. The obtained results show that the light coupling efficiency increased to 77.8%. Plasma surface treatment was used to immobilize gold nanoparticles (AuNPs) on the tip of the POF, as a LSPR-POF sensor. Adsorption kinetics was measured based on the pseudo-first-order model to determine the efficiency of immobilizing AuNPs, in which the adsorption rate constant (k) was obtained be 8.6 × 10^–3^ min^−1^. The RI sensitivity of the sensor in the range from 1.3332 to 1.3604 RIU was obtained as 7778%/RIU, and the sensitivity was enhanced ~ 5 times to the previous RI POF sensors. These results are in good agreement with theory and computer simulation. It promises a highly sensitive and label-free detection biosensor for point-of-care applications such as neurosciences.

## Introduction

The scattering and darkness of the tissue have limited the penetration of light in it. In the past few decades, optical fiber has been used to provide light to different areas of the body^1^. Fiber optic sensors (FOSs) are currently garnering significant interest and quickly making their way into market^2^. Sensors made using silica fiber optics often have limitations and a short lifespan. Their low biocompatibility and lack of flexibility have led to one of the most challenging issues related to fiber optic sensors. Silica optical fibers are rigid and brittle^3^. The high stiffness of conventional optical fiber may easily cause tissue damage during implantation or movement^4^.

Polymeric optical fibers (POF) have unique properties such as flexibility, biocompatibility, and biodegradability. Furthermore, POFs have a low loss window in the visible wavelength, making them an ideal choice for sensing applications. These features can be easily used in biomedical applications for implantation in the body. Table 1 provides a summary of the various types of POF that have been developed. This research used citrate-based polymers (CBP) with high biocompatibility, degradability, and flexibility. Additionally, CBP optical fibers exhibit minimal losses due to their dual-component design comprising a core and a cladding, making them suitable options for constructing optical fiber sensors.

**Table 1:** Materials, structures, optical parameters, application, and year of typical optical waveguides.

Materials	Structure	Optical parameter	Application	Ref
Hydrogels (PEG)	Single- Layer	491 nm	Optical sensing	^1^
Silicone rubber	Core- Cladding	530–685 nm	Sensor	^5^
polydimethylsiloxane (PDMS)	Core- Cladding	480–1000 nm	UCNPs-SPOFs sensor	^6^
PLLA polymer	Single- Layer	473 nm	Deliver light	^7^
Poly (ethylene glycol) di acrylate (PEGDA)	Core- Cladding	490 nm	Fluorescence Sensor	^8^
PEG-diacrylate (PEGDA)	Core- Cladding	560 and 640 nm	Deliver light and photothermal	^9^
Silk hydrogel	Single- Layer	540 nm	Deliver light	^10^
Poly (octamethylene citrate) (POC) Poly (octamethylene maleate citrate) (POMC)	Core- Cladding	633, 532and 473 nm	Deliver light and imaging	^11^ & this work

Due to its multimodal characteristics, a POF-based refractive index (RI) sensor is well-suited for intensity modulation schemes^12^. Several solutions can be combined to improve the sensor's sensitivity, such as adjusting the shape, working wavelength, and surface modification. When using visible light, variations in light intensity due to different RI values are easily noticeable and detectable^13^.

**Figure 1:**
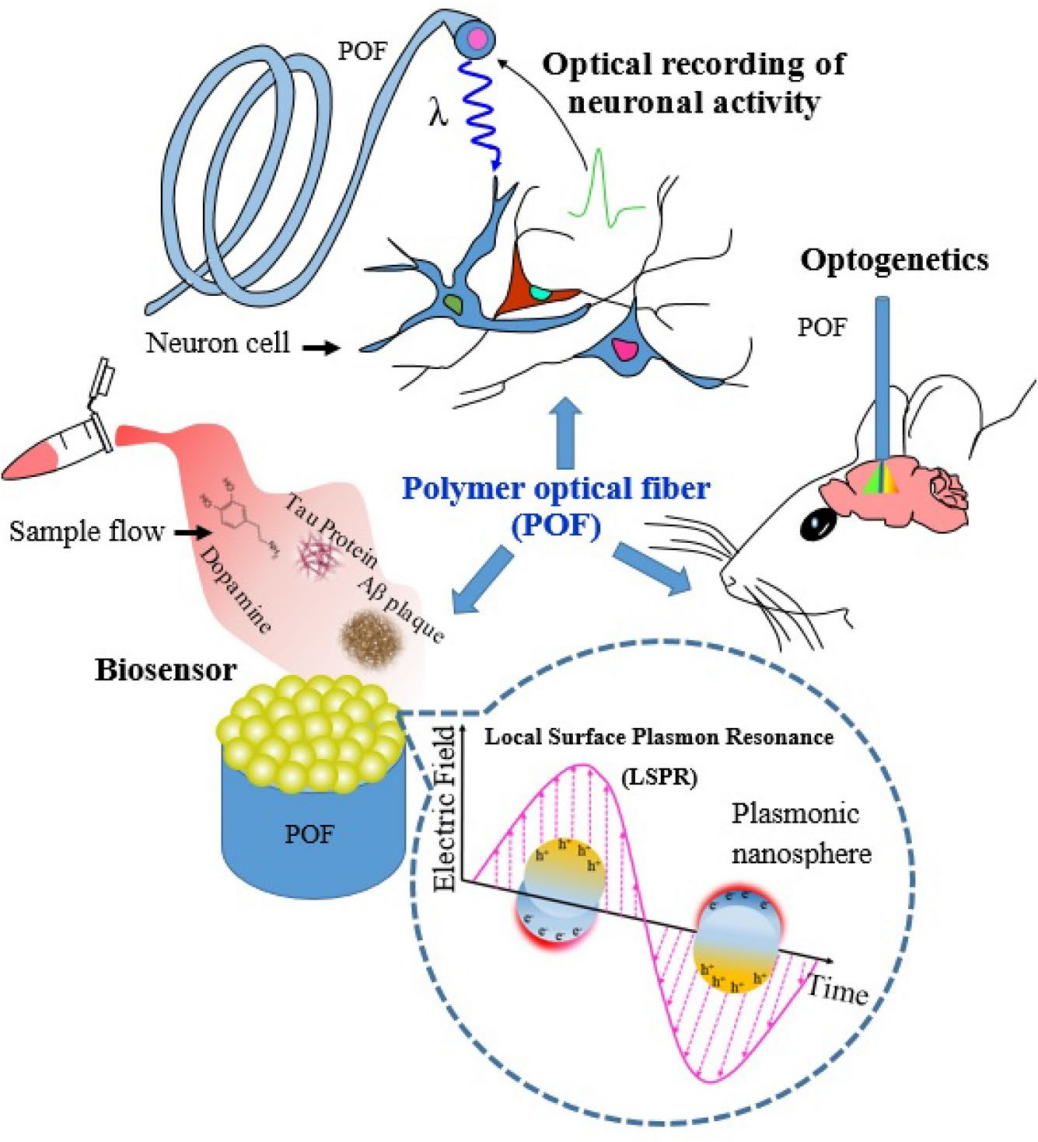
Applications of POF sensors in neurophotonics.

Label-free fiber optic biosensors (FOBs) based on RI are desirable^2^. Combining FOBs with nanotechnology can significantly improve the sensor's sensitivity. Localized surface plasmon resonance (LSPR) is a popular method for label-free biosensing, as it allows for sensitive, robust, and easy detection of biomolecular interactions. LSPR-based biosensing works by detecting changes in the local RI at the surface of a nanoparticle (NP), affecting the plasmon frequency. This technology enables precise measurement of environmental changes^14–16^. Localized surface plasmons (LSPs) are oscillations of charge density that occur within metal nanoparticles. When light excites LSPs in metallic NPs, it produces scattering and absorption, which can be utilized to detect and characterize molecules and materials near to the metallic NPs. The change in transmitted intensity is measured by monitoring the absorbance spectra or a wavelength shift in the peak absorbance, indicating a change in the surrounding environment^17^. The ability of LSPs to concentrate electromagnetic fields in the vicinity of the NPs enables the enhancement of the interaction between light and matter, leading to increased sensitivity and selectivity in detection and imaging techniques. Figure 1 shows the schematic of some POF applications in neurophotonics, such as optogenetics stimulation^18^, biosensors, and optical neural activity recording.

This study presents a soft and flexible RI-POF sensor based on LSPR. The optical sensor was made of a biocompatible POF incorporating electrostatically immobilized gold nanoparticles (AuNPs). POF’s low transmission efficiency poses a significant challenge in the fabrication process. Some POFs exhibit poor light transmission efficiency because they lack cladding and suffer from improper light coupling inside them^3,4,19^. In previous studies, lenses and blades were used for light coupling to the POF and cutting, respectively^11^. Unlike previous literature, this study uses microsphere fiber optics to enhance the coupling efficiency. Besides, a new chemical method has been used to cut the tip of POF to reduce the surface roughness caused by blade cutting. Accordingly, efficiency has increased significantly by using these two methods for the first time. During the immobilization of AuNPs, the adsorption kinetics were studied as a key characteristic for sorption efficiency. The present results showed that the kinetic model followed a pseudo-first-order, indicating that the adsorbent and adsorbate interactions were due to van der Waals forces. In this investigation, the potential of an LSPR-POF sensor was analyzed based on a reflection mode set-up using intensity interrogation for the RI measurement. The sensor advantages include low cost, high sensitivity, and easy detection. This sensor can be a good candidate for detecting different biomarkers in neurodisoders^20^, such as Alzheimer's, Parkinson's, and the like, and therapeutic drug monitoring (TDM)^21^ applications.

## Materials and methods

### Reagents

Further processes were not applied to analytical grade chemical substances.1,8-Octanediol (98%) and gold (III) chloride trihydrate (HAuCl_4_.3H_2_O) were purchased from (Sigma Aldrich), citric acid and trisodium citrate were purchased from (Bio basic, Canada INC), Maleic anhydride,1,4 Dioxane, and Ethanol (30%), and double-distilled water were purchased from (Merck).

### Synthesis of polymers

POF was made based on poly (octamethylene maleate citrate) POMC as the core and poly (octamethylene citrate) POC as the cladding. The synthesis process was performed according to the Yang method^22,23^ with slight modifications. In order to synthesize the POC prepolymer, a combination of citric acid and 1.8-octanediol in a 1:1 molar ratio was heated at 160 °C for 20 min, followed by an additional hour at 140 °C. To prepare the POMC prepolymer the researchers employed a mixture of citric acid, maleic anhydride, and 1.8-octanediol in a molar ratio of 0.4:0.6:1. The heating process involved melting the mixture at 160 °C for 20 min, followed by a continuation at 140 °C for 1 h to achieve the desired POMC prepolymer.

### Synthesis of NPs

**Figure 2:**
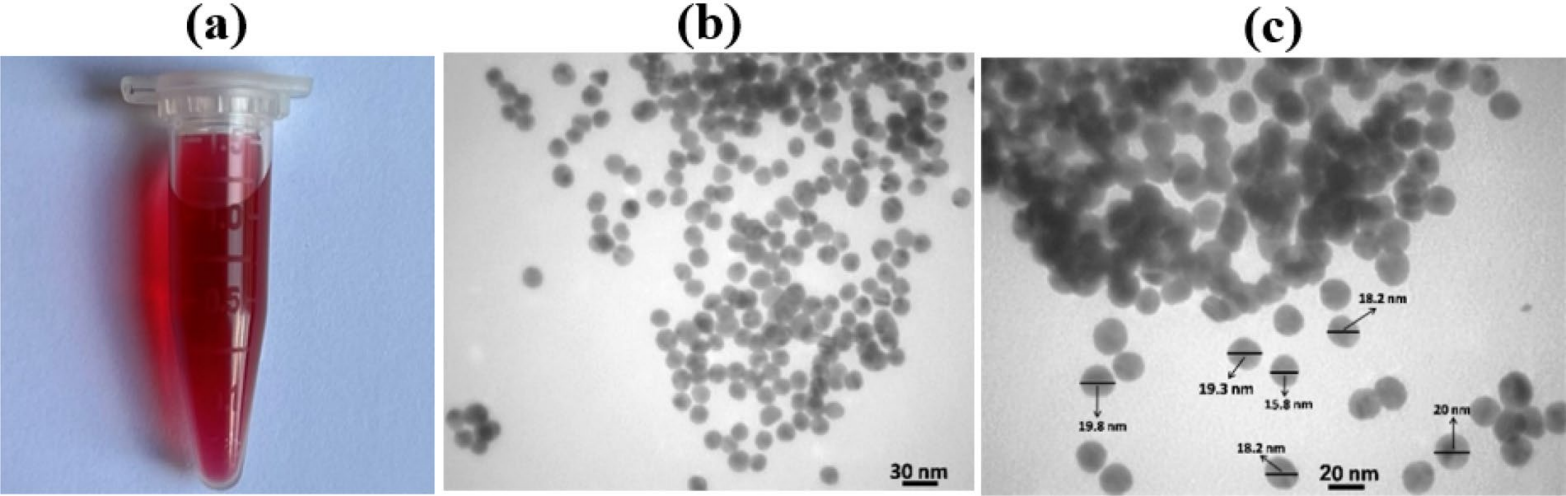
(**a**) The appearance of AuNP solution. (**b**, **c**) TEM image of synthesized NPs.

The synthesis of AuNPs involved using the citrate reduction method. This process commenced by adding 3mL of HAuCl_4_.3H_2_O to a 1-L round bottom flask, followed by elevating its temperature to 90 °C. Then, 2.5 mL of trisodium citrate (0.2 M) solution was added and mixed under vigorous stirring. Ultimately, reflux continued for 10 min until the solution’s color changed from yellow to ruby red. The prepared solution can be stored at 4 °C for a few months. Figure 2a shows the synthesized AuNPs solution. Figure 2b shows the transmission electron microscopy (TEM) images in Fig. 2b,c, representing that the NPs are uniform with a size of about 20 nm.

### Synthesis method and characteristics investigation of POC and POMC

This study evaluated two elastomers, citrate-based poly (octamethylene citrate) (POC) and poly (octamethylene malitrate) (POMC), for the development of a flexible and degradable biocompatible step-index POF.

Fourier transform infrared (FTIR) spectroscopy and differential scanning calorimetry (DSC) measured the chemical properties of POC and POMC polymers. For this purpose, the POC prepolymer solution was placed on the glass slide for 7 days at 70 °C and then 3 days at 80 °C. In the case of the POMC prepolymer, the solution was placed in an oven for 3 days at 70 °C and then at 80 °C for 3 days. These incubation periods led to the completion of the polymerization reaction^11^.

**Figure 3:**
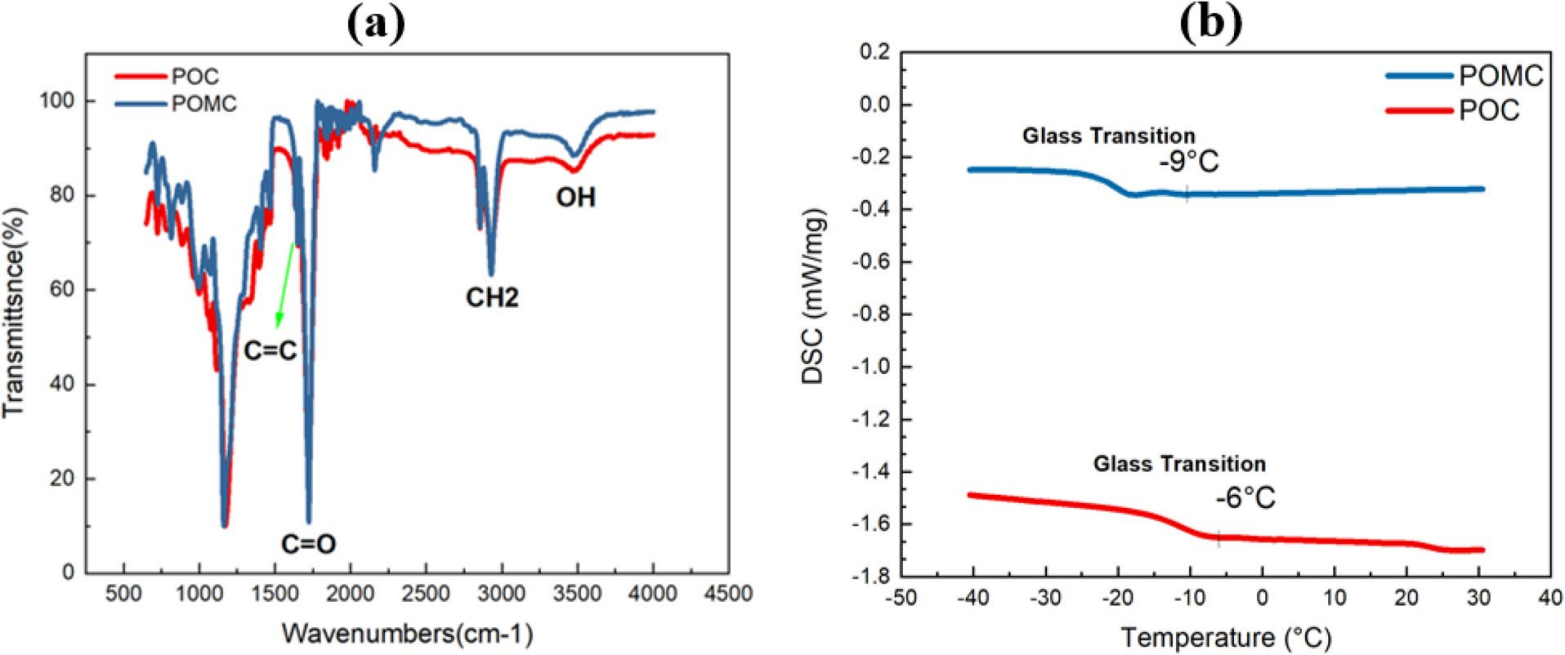
(**a**) FTIR spectra of POMC and POC. (**b**) Thermal characterization of POMC and POC films, DSC thermograph with respective Tg at − 9 °C and − 6 °C.

The characteristic FTIR spectra of the POMC and POC reactions are shown in Fig. 3a. The functional groups have been confirmed according to reports^24,25^. To determine the amorphous region of the polymer, an analysis of its thermal properties was conducted through DSC testing. For the analysis of POMC using DSC, the first step involved scanning the samples up to 150°C at a heating rate of 10 °C/min in nitrogen to remove any traces of water. The samples were then rapidly cooled at a cooling rate of 40 °C/min to − 60 °C. A second scan was performed up to 230 °C at a heating rate of 10 °C/min^24^. For POC analysis, the scanning rate was determined as 10 °C/min, and the sample was heated from − 50°C to 200°C under a nitrogen atmosphere^26^. According to Fig. 3b, the results showed that in the DSC spectra of POMC and POC polymers, the glass transition temperature (T_g_) is – 9 °C and – 6 °C, respectively. POMC and POC polymers' glass transition temperature values are below 0°C. The obtained results indicated that polymers retain their amorphous, flexible, and soft properties at body temperature^24^. The polymer's amorphous structure makes it transparent, allowing it to transmit light when implanted in the body.

### Polymer degradation study

**Figure 4:**
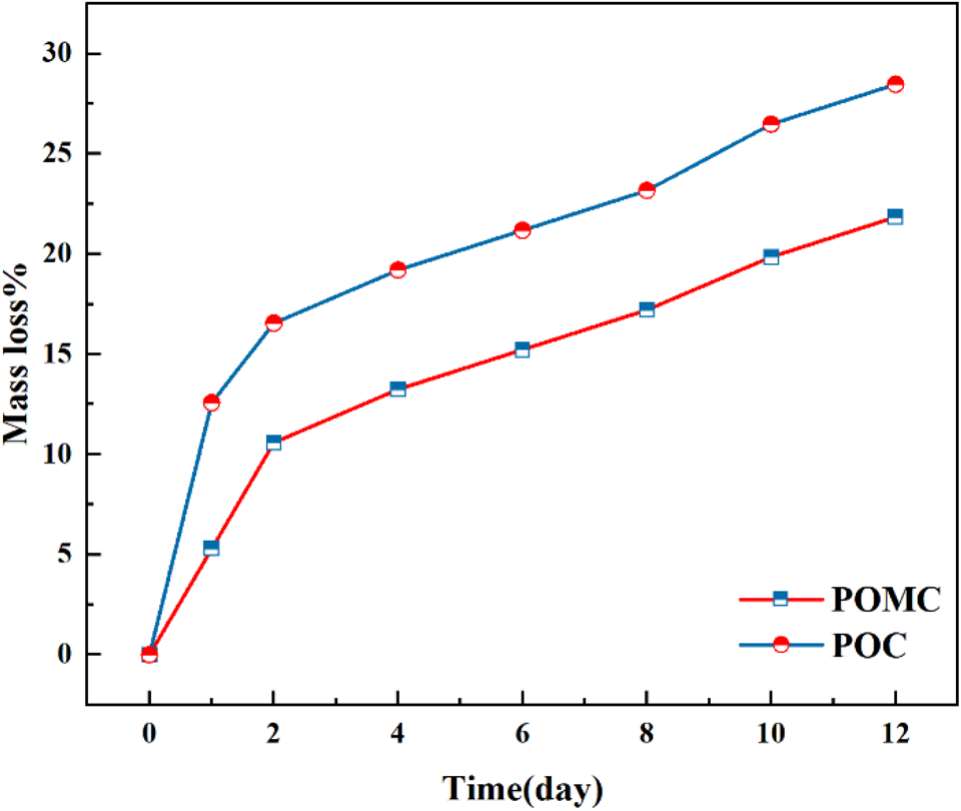
In-vitro degradation of POMC and POC in PBS (pH 7.4, 37◦C).

Each 5 mm × 5 mm in size was cut and weighed to find their initial mass to measure the degradability of POMC and POC polymers. The polymers were placed in phosphate buffer saline (PBS) at pH 7.4 and incubated at 37 °C for 12 days. The buffer was added every two days to maintain a constant pH of 7.4. At the desired time, the samples were washed in de-ionized water, dried at ambient temperature, and weighed to find their mass. According to Fig. 4, the results showed that during 12 days, polymer POC and POMC lost 27% and 22% of their polymer mass, respectively. The results demonstrated that the core and cladding polymer exhibit consistent degradation rates over the course of 12 days. This implies that they disintegrate simultaneously within the biological environment. Additionally, an NaOH solution was utilized in the investigation to hasten the degradation process. The polymers were placed in 0.1 M NaOH solution and maintained at 37 °C for 12 h.

### Fabrication of optical *fiber* microsphere

**Figure 5:**
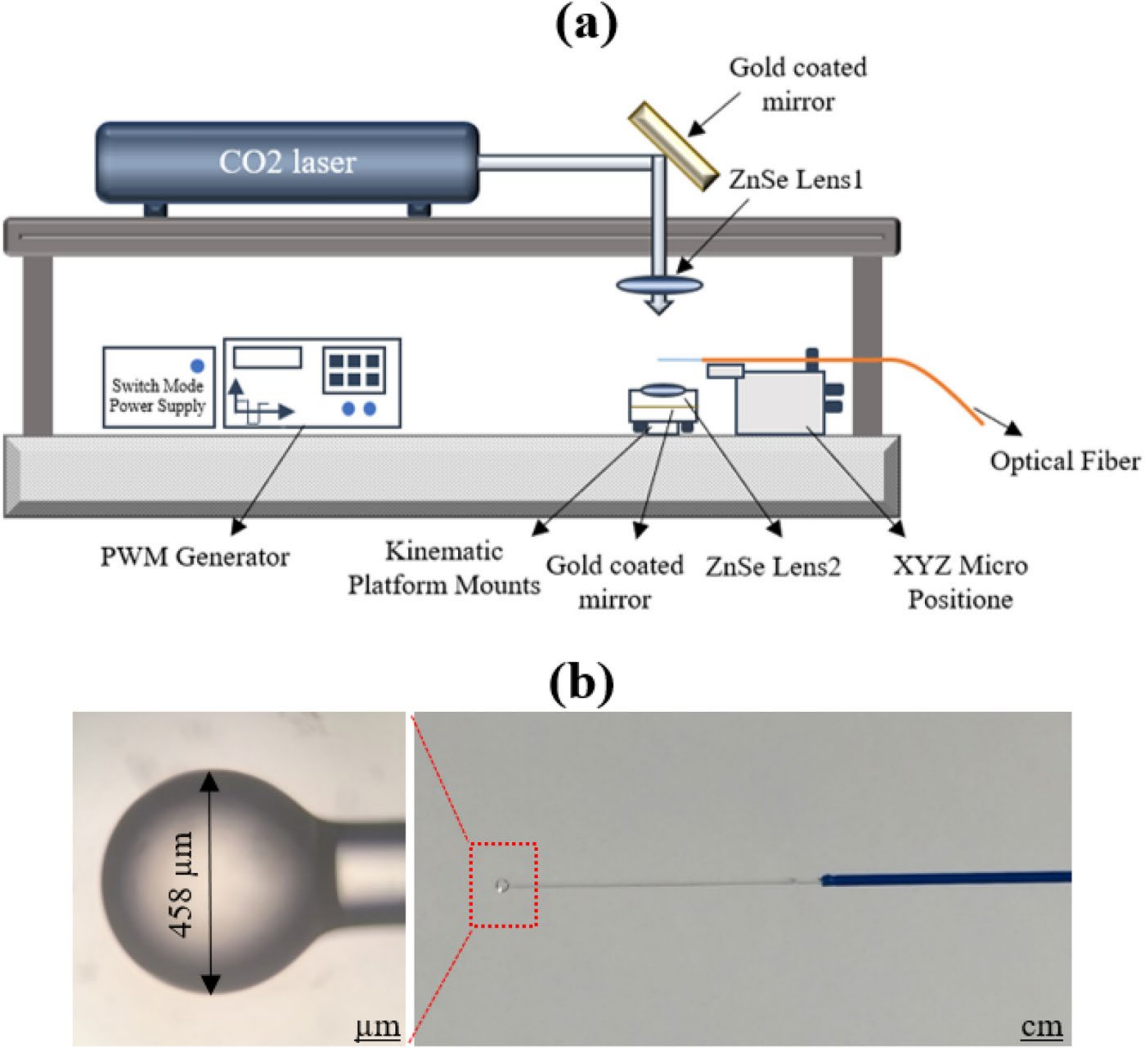
(**a**) Schematic of microsphere fabrication process using CO_2_ laser. (**b**) A typical image of fabricated microsphere on tip of fiber optic.

A microsphere was fabricated as a spherical lens at the tip of optical fibers to enhance light coupling to the POF. During the POF fabrication process, the microsphere was embedded in the POF. The microsphere was fabricated using a CO_2_ laser by applying heat to the tip of a multimode fiber optic with a numerical aperture of 0.39 with core and cladding diameters of 200 μm and 250 μm, respectively. Figure 5a shows the schematic of laser heating in which the laser beam was focused through a Zn-Se lens (L1) with a focal length of 2.5 cm onto the fiber tip. After colliding with the optical fiber, the laser beam reaches the second Zn-Se lens (L2), and a gold-plated mirror is embedded below the lens (L1). The reflected beam is refocused on the fiber tip^23^. The device's frequency was set to 5 kHz, the number of pulses was 50,000, and the duty cycle was 90%. The fabricated microsphere, with a diameter of 458 μm, is shown in Fig. 5b.

## Simulation results

### LSPR–POF simulation

Surface plasmons are the collective oscillations of electrons in the thin layer of a metal surface. These oscillations can generate polaritons, often referred to as surface plasmon polariton (SPP) modes. The surface plasmon, excited by incident light on the thin metal layer, can emit light in all directions (polariton), with its optical intensity peak at the plasma frequency^27^. When the core mode is coupled to the SPP mode, all of the evanescent fields of the core mode penetrate the metal layer, and the propagation constants of the SPP mode (β_SPP_) match with the core mode (β_Core_)^28,29^.

1 $${\beta }_{SPP}=\frac{\omega }{c} \sqrt{\frac{{\varepsilon }_{m} {\varepsilon }_{ex}}{{\varepsilon }_{m}+ {\varepsilon }_{ex}}}\quad \& \quad{\beta }_{Core}=\frac{\omega }{c} \sqrt{{\varepsilon }_{ex}}\text{ sin}{\theta }_{0}$$

where ω, c, ${\varepsilon }_{m}$, and ${\varepsilon }_{ex}$ are the angular frequency, speed of light, and the real part of relative permittivity of the thin metal layer and the external medium, respectively. According to Eq. (1) ${\beta }_{spp}$ depends on the RI of the external medium, which is the basis of RI sensing using SPP^27,29^.

In the visible and the near-infrared wavelength range, the Drude free electron model alone cannot interpret the dielectric function of the metal because the bound-interband electron oscillation effect described by the Lorentz model is noticeable. Considering that the SPP excitation of Au often occurs continuously in the visible and near-infrared regions, the complex relative permittivity ${\varepsilon }_{c}$ of metals according to the Drude-Lorentz model is according to^30^:

2 $${\varepsilon }_{c}={\varepsilon }_{c}^{(D)}(\omega )+{\varepsilon }_{c}^{\left(L\right)}(\omega )$$

3 $${\varepsilon }_{c}^{\left(D\right)}\left(\omega \right)=1-\frac{{\Omega }_{p}^{2}}{\omega (\omega -i{\Gamma }_{0})}$$

4 $$\varepsilon_{c}^{\left( L \right)} \left( \omega \right) = \mathop \sum \limits_{j = 1}^{k} \frac{{f_{j} \omega_{p}^{2} }}{{\left( {\omega_{j}^{2} - \omega^{2} } \right) + i\omega \Gamma_{j} }}$$

Where $\varepsilon_{c}^{\left( D \right)} \left( \omega \right)$ and $\varepsilon_{c}^{\left( L \right)} \left( \omega \right)$ are complex relative permittivity of the metal layer based on Drude and Lorentz model, respectively.$\omega_{p}$ is the plasma frequency of free electrons, $\Omega_{p} = \sqrt {f_{0} \omega_{p} }$ is the bound-interband electron plasma frequency that oscillates with oscillation strength ${f}_{0}$ and damping coefficient ${\Gamma }_{0}$, *k* is the number of bound-interband electron oscillators that oscillate with frequency ${\omega }_{j}$ and power ${f}_{j}$ and lifetime $\frac{1}{{\Gamma }_{j}}$.

The most important factor that SPP modes suffer from is loss. This includes the attenuation of the emitted polaritons, reduced electron confinement at the metal-environment boundary, surface roughness above the fiber-metal coating, and metal heat loss^31,32^. One of the unique tricks that enables the photonic structure to overcome these limitations of plasmonic loss is using LSPR mode instead of SPR mode^33^. The loss coefficient for resonant media can evaluate the degree of coupling between the fiber core mode and the SPP mode. When the maximum coupling between the core mode and the LSPR mode occurs at that wavelength, the loss coefficient α is at its maximum, which is defined as follows^33^:

5 $$\alpha_{Loss} \left( \lambda \right) = \frac{40\pi }{{\lambda (\ln 10)}} {\text{Im}} \left( {n_{eff} } \right) \left( {\frac{dB}{{Cm}}} \right)$$

6 $$n_{eff} = \sqrt {\varepsilon_{c} \varepsilon_{ex} /\left( {\varepsilon_{c} + \varepsilon_{ex} } \right)}$$

where ${\text{Im}}\left({\text{n}}_{\text{eff}}\right)$ is the imaginary part of the effective RI that depends on the external medium RI of the fiber. Therefore, changing the external medium's concentration changes the LSPR signal's peak intensity (both reflection and transmission)^34^.

**Figure 6:**
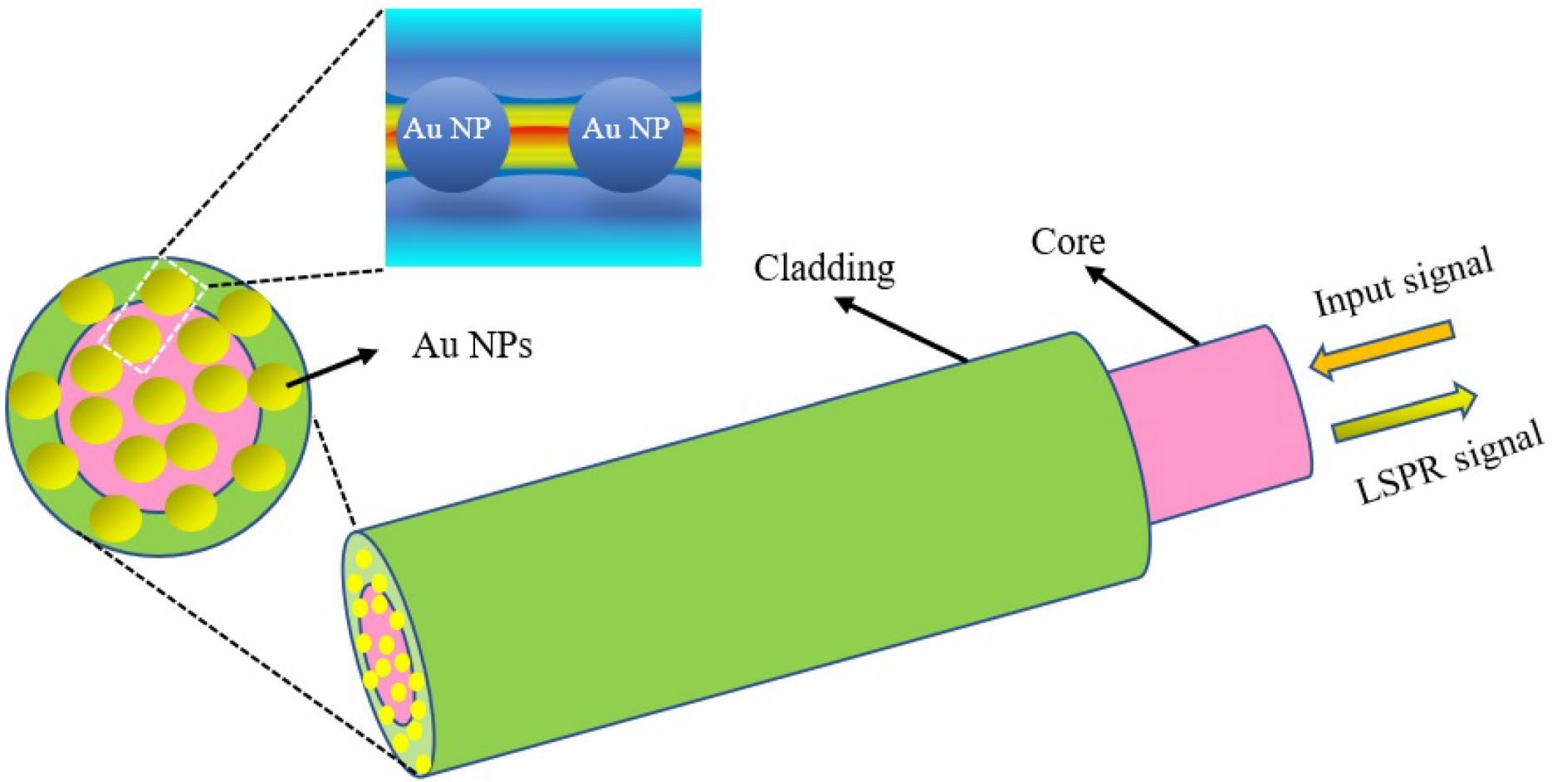
Schematic of the proposed POF which AuNPs were immobilized to the tip.

The simulation was accomplished based on the finite element (FEM) method using the electromagnetic waves beam envelopes (EWBE) module and boundary mode analysis physics. Due to the significant size disparity between the proposed POF structure and the AuNPs, the outcome of the simulation is contingent upon the proper meshing of the structure (with a maximum element size on the order of λ/10), resulting in prolonged computational duration. This study used structural symmetry and periodic boundary conditions to solve this problem^32,35–39^. The present study only simulated that part of the POF structure, including 2μm of the clad area and 2μm of the core area. AuNPs with a diameter of 20 nm were arranged on the POF tip. A schematic of the LSPR-POF structure is shown in Fig. 6.

The RIs of the core and cladding POF at the plasmonic resonance wavelength λ_res_ = 625 nm were n_core_ = 1.498 RIU and n_clad_ = 1.492 RIU, respectively. Because of this RI difference, optical modes whose effective RIs satisfy the condition *n*_*clad*_ < *n*_*eff*_ < *n*_*core*_ are propagated with minimal losses in the polymer core of the fiber.

**Figure 7:**
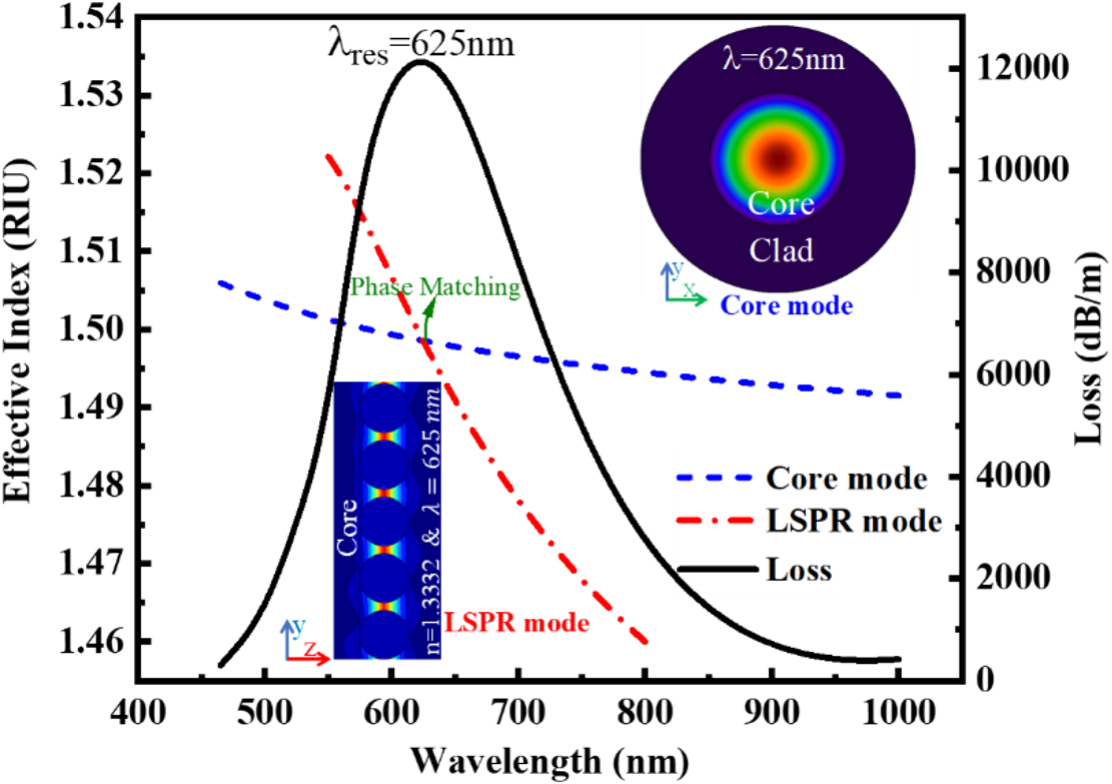
The loss coefficient curve with the effective RI of core mode and the LSPR mode according to different wavelengths for external medium RI of n = 1.3332 RIU.

**Figure 8:**
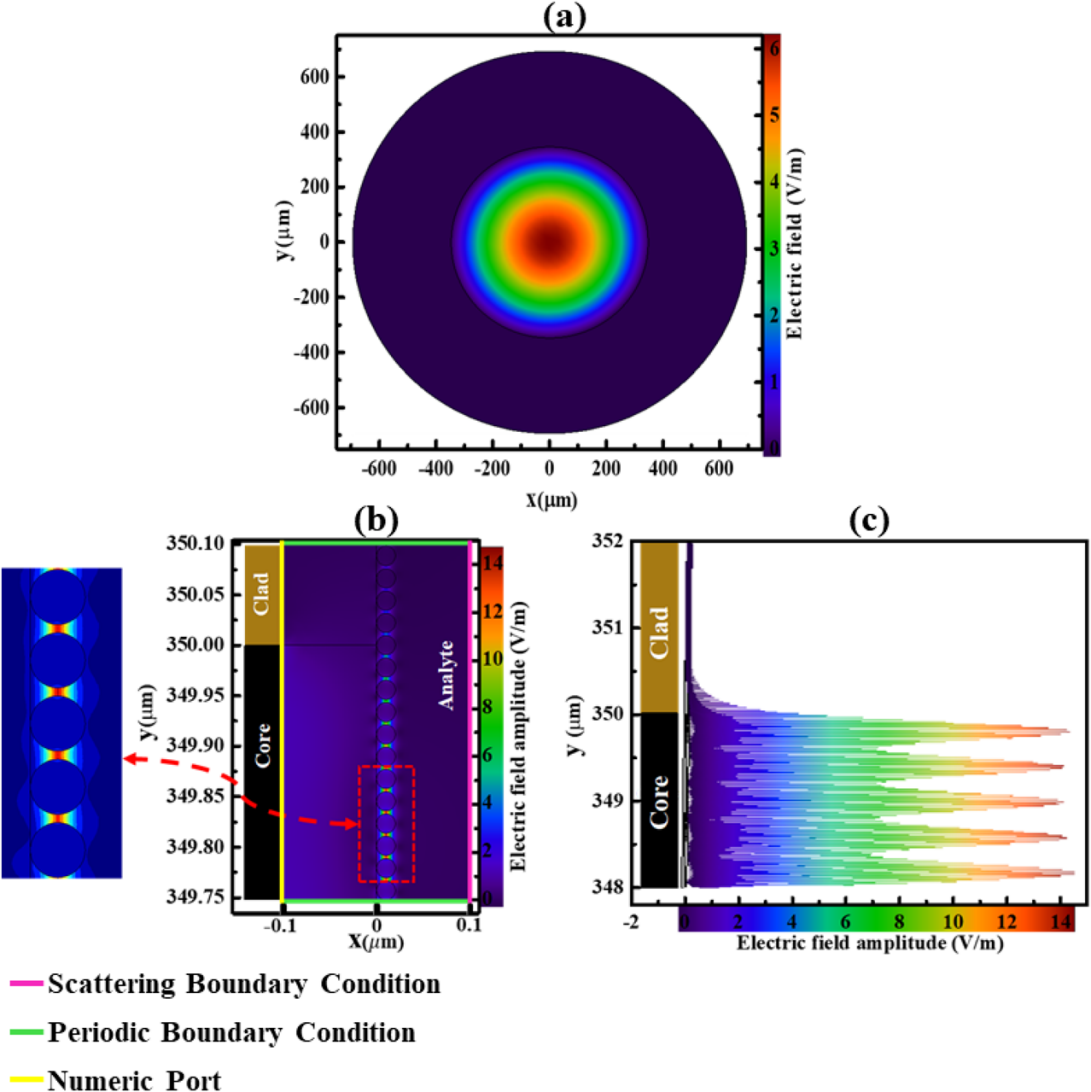
(**a**) Electric field distribution (V/m) of Core mode of POF from the tip cross-section (x,y). (**b**) Electric field distribution (V/m) of AuNPs immobilized on POF tip at fiber air interface for the Cross-sectional (y,z) with considering scattering and periodic boundary. (**c**) Longitudinal (y) view. Finite element analysis of POF, showing the EM field variation excited at the wavelength of 625 nm and RI of bioanalyte n = 1.3332 RIU.

When the effective RIs of the core and LSPR modes were equal, the loss coefficient reaches its maximum value, showing the maximum coupling between these modes^33^. Based on the simulation result shown in Fig. 7, when the RI of the external medium is equal to 1.3332 RIU at the wavelength λ = 625 nm, where the real part of effective RI of core and LSPR modes were equal to n_eff_ = 1.4985 RIU, the phase matching happens, and the maximum loss coefficient α_loss_ = 12,125 dB/m has been obtained. The electric field profile of these two cores and LSPR modes is shown in Fig. 8.

**Figure 9:**
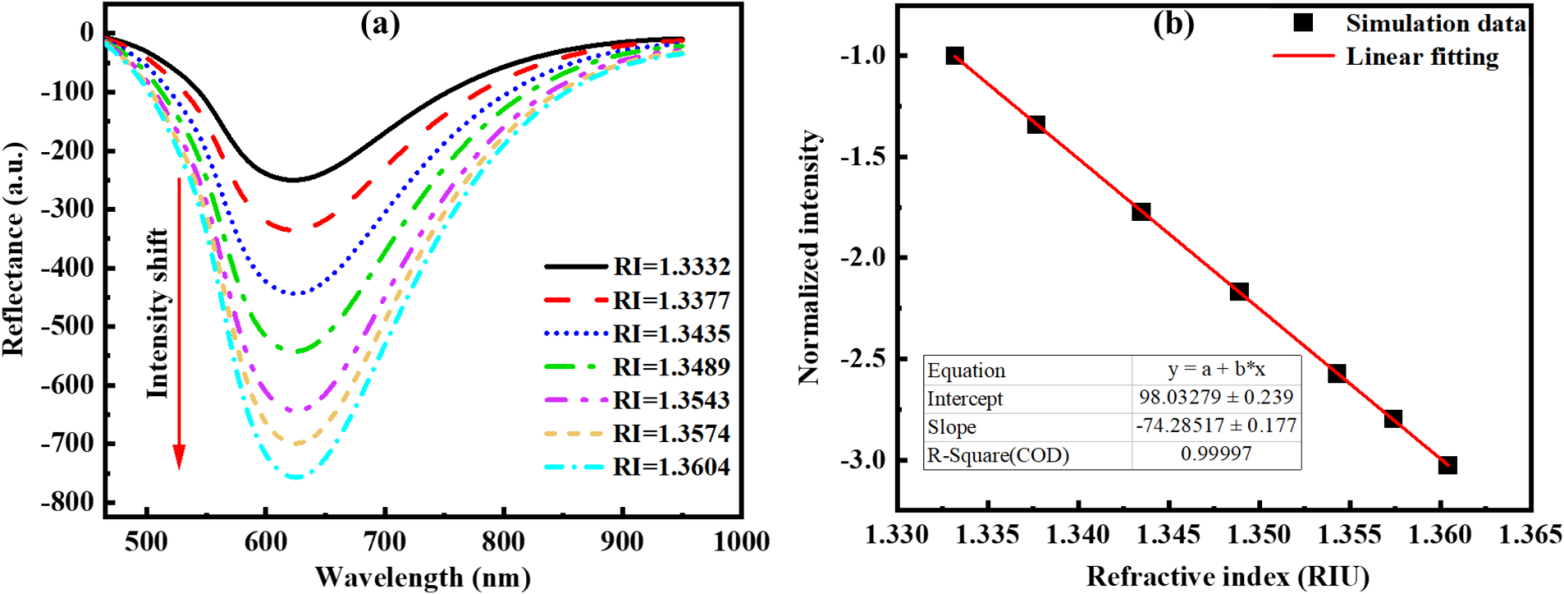
(**a**) Changing reflected LSPR spectrum of the sensor in different Ris. (**b**) Obtained RI sensitivity of LSPR–POF sensor.

According to Fig. 9a, the simulation results obtained showed that changing the RI from 1.3332 to 1.3604 RIU led to intensity changes in the reflected spectrum of the LSPR-POF sensor. Figure 9b shows that the RI sensor's sensitivity was obtained to be − 7428.5%/RIU with R-Square 0.998.

### *Fiber* optic microsphere size optimization

**Figure 10:**
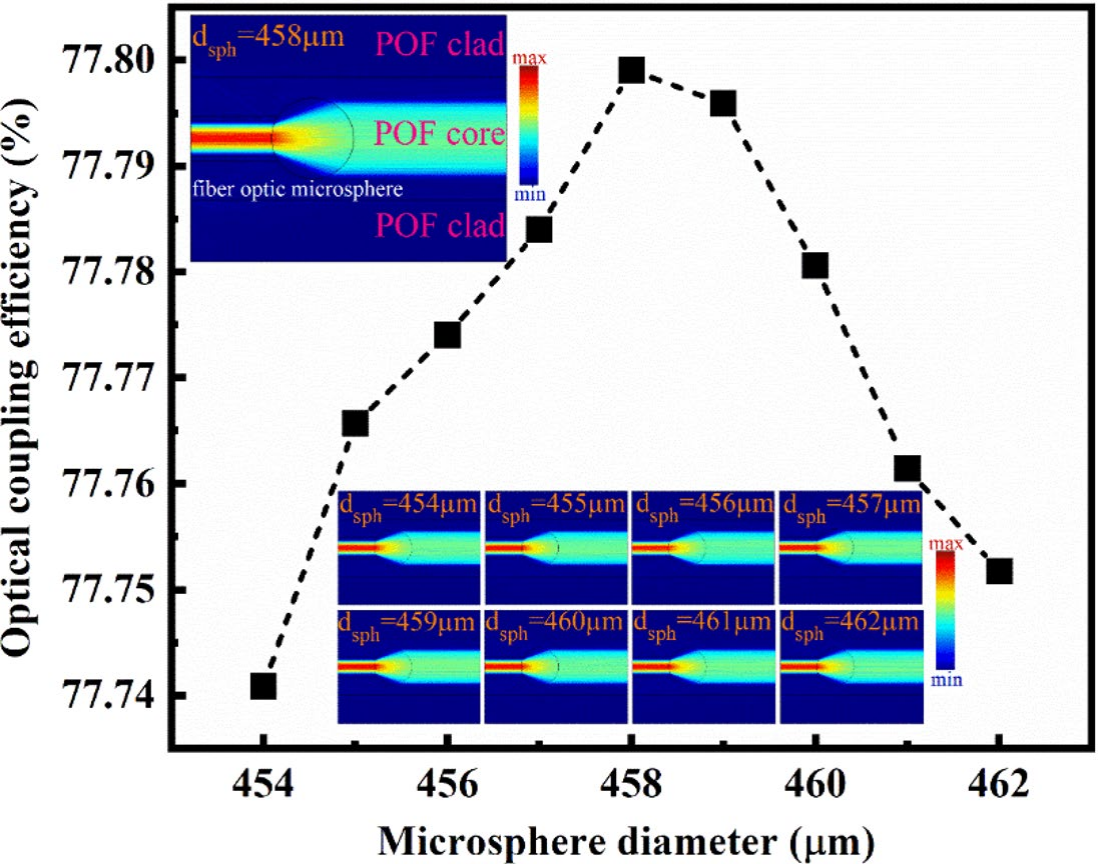
Optical coupling efficiency of light to the POF versus the microsphere diameter at 625 nm.

The fiber optic microsphere is a unique photonic structure for light coupling with high efficiency in the optical fiber core, and its diameter significantly affects the light coupling efficiency of this optical coupling^40^. Furthermore, optical coupling efficiency affects RI sensitivity by enhancing the signal-to-noise ratio (SNR) of measurement in the intensity modulation method. Simulation results based on FEM, as shown in Fig. 10, indicate that the maximum coupling efficiency microsphere to the POF at the wavelength λ = 625nm with microsphere in diameter *d*_*sph*_ = *458 µm* was 77.8%.

## Results and discussion

### Design and Fabrication of LSPR sensor

#### Fabrication of step-index fibers with microsphere

Previous studies utilized a lens to couple light within an optical fiber^11^. This approach leads to light loss during its transmission to the optical fiber, consequently diminishing the fiber optic's efficiency. This research addressed this issue using a microsphere as a coupling lens to couple the light to the POF.

**Figure 11:**
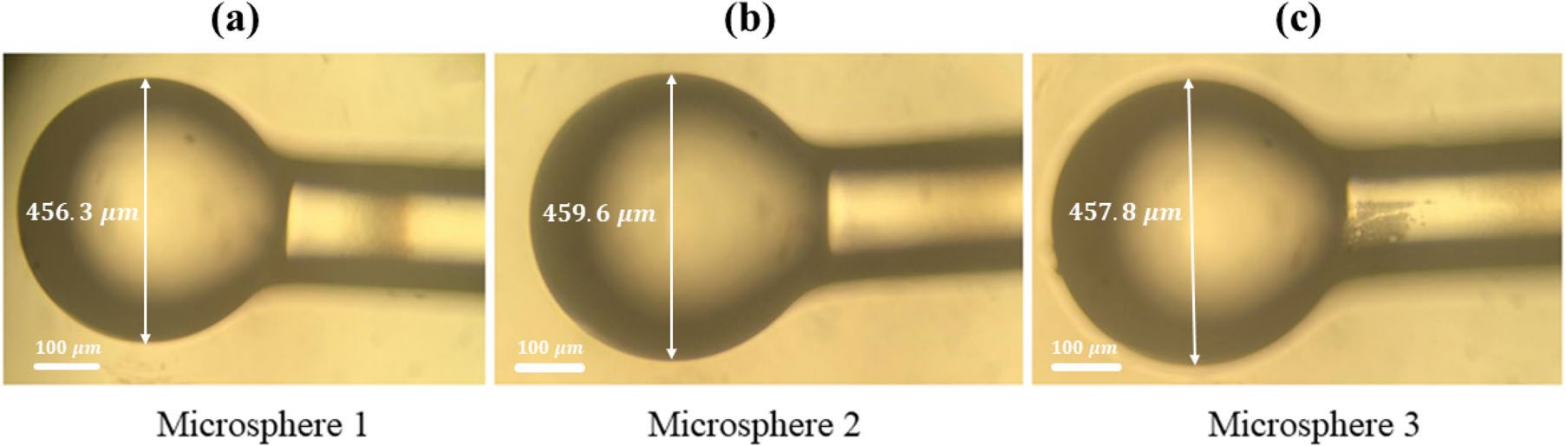
Typical images of different manufactured microspheres with a diameter (**a**) 456.3 µm, (**b**) 459.6 µm, and (c) 457.8 µm.

**Figure 12:**
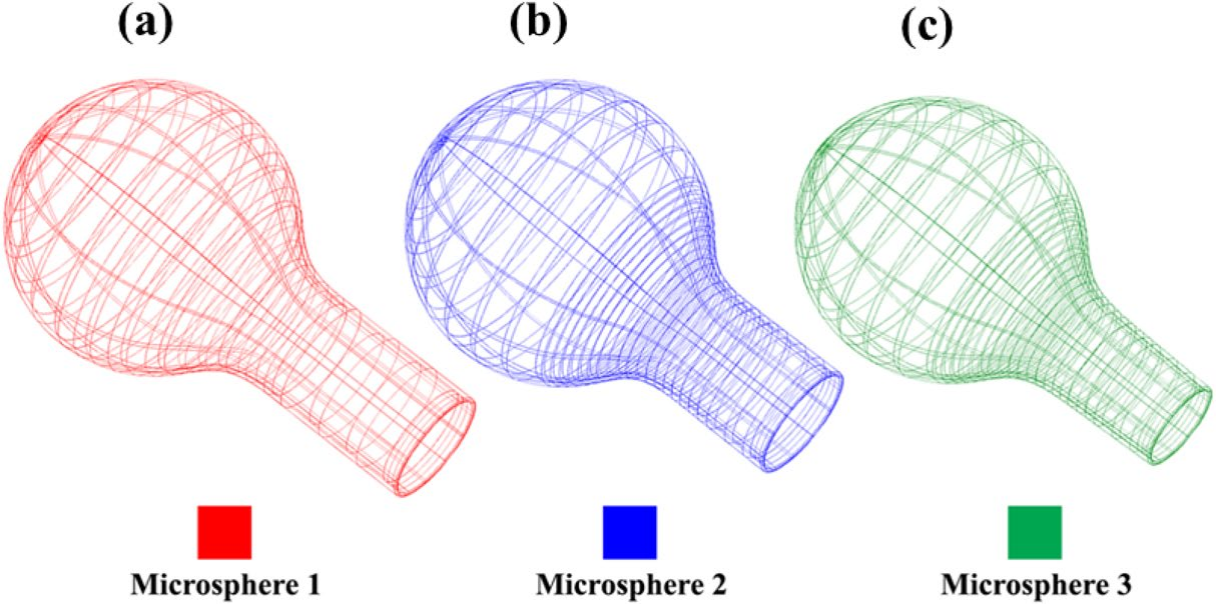
3D profiles of different manufactured microspheres, as shown in Fig. 11.

During the manufacturing of microspheres, a certain length of optical fiber is exposed to the laser to make microspheres. The precision of this process is unparalleled, with all parameters affecting the size of microspheres being adjustable and remaining constant. This ensures the production of microspheres of the same size, as depicted in Fig. 11. The three-dimensional image processing of each microsphere has been illustrated in Fig. 12, further demonstrating the meticulousness of our approach.

**Figure 13:**
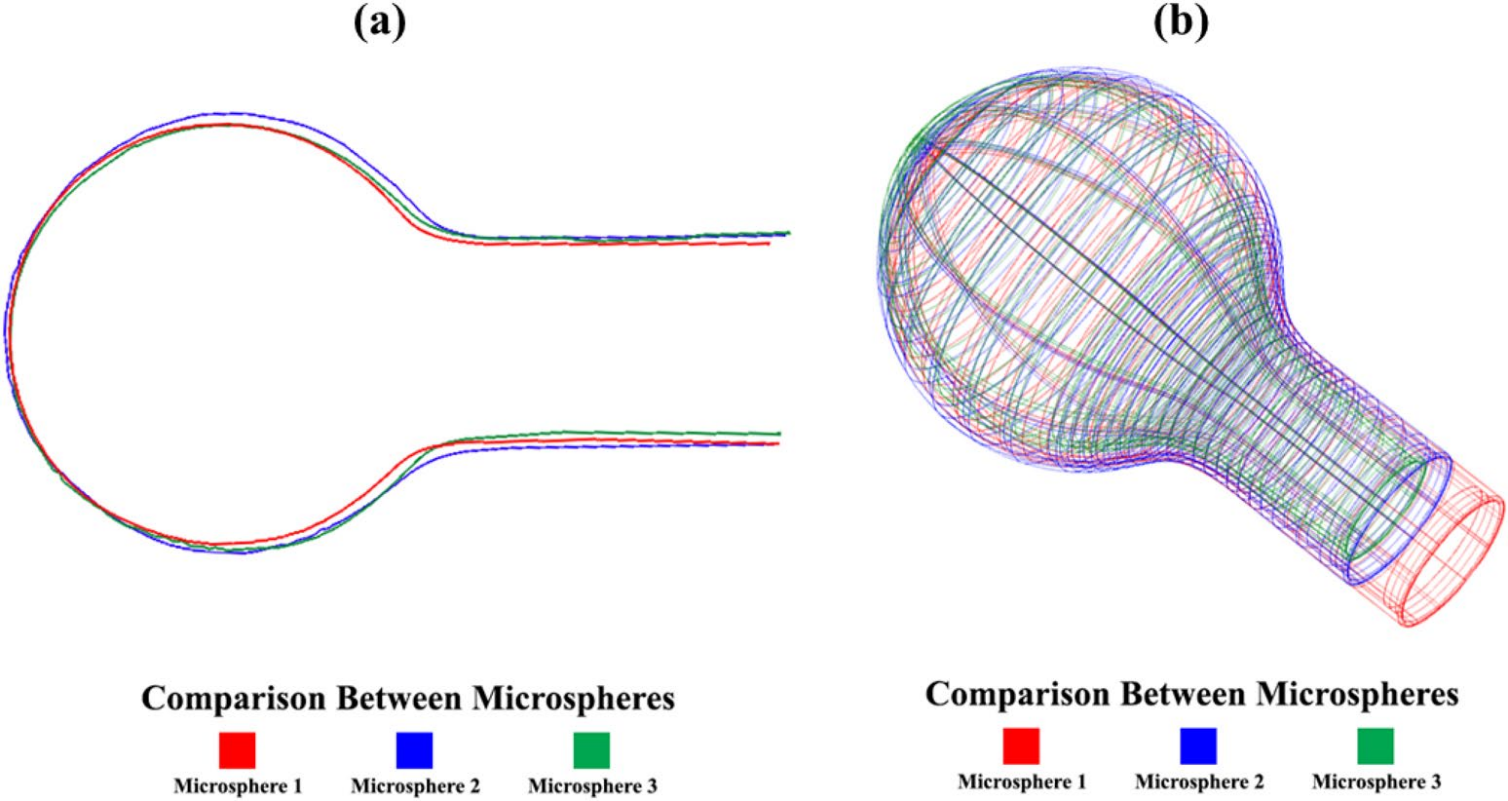
Comparing different profiles of micropheres, (**a**) 2D profiles and, (**b**) 3D profiles.

Figure 13 compares different profiles of the fabricated microspheres, having suitable fabrication repeatability and a diameter of about ~ 458 ± 2µm.

**Figure 14:**
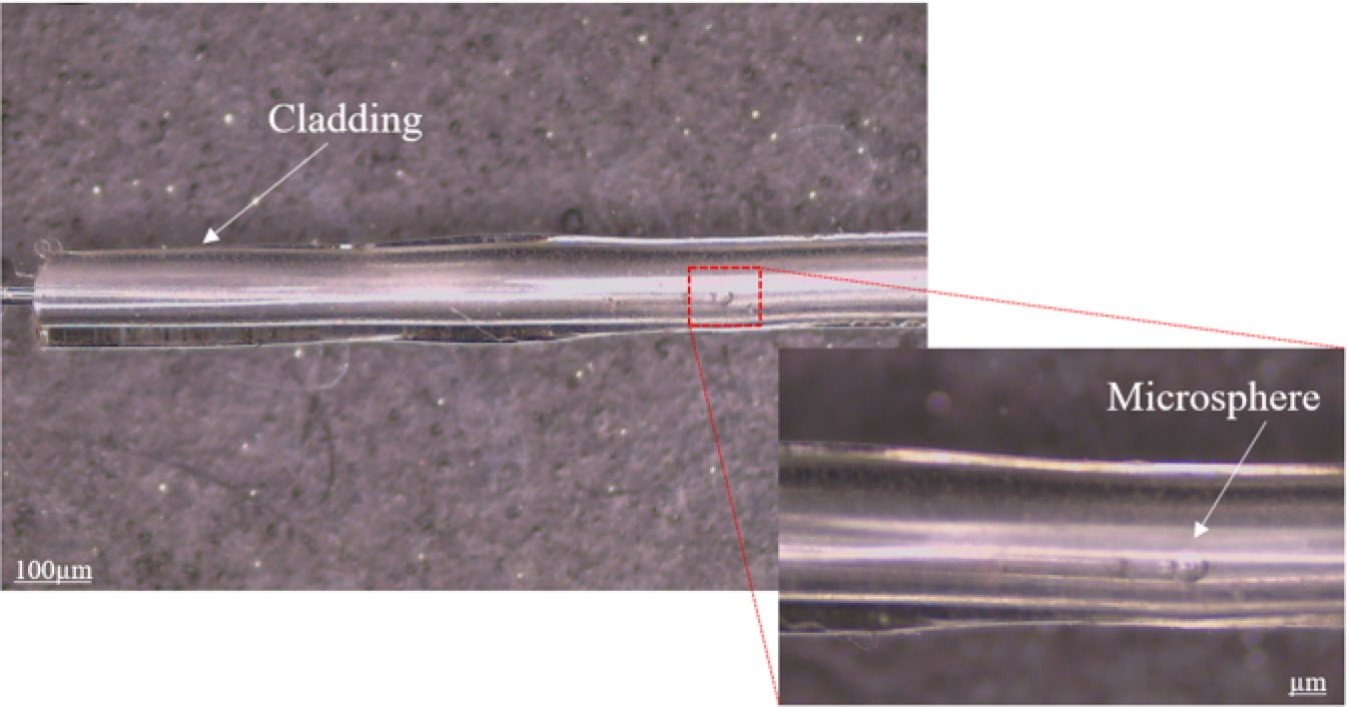
Microsphere inside POMC tube as optic fiber cladding.

A two-step manufacturing method has been used to develop an optical fiber to create a two-layer structure of cladding and core. In the first step, the cladding layer was created by dip-coating a stainless-steel wire with a diameter of 700 µm into the POC prepolymer liquid and heating it at 70°C for 4 days. Then, to remove the wire from the coating layer, the wire was immersed in a 30% ethanol solution for 1 day. In the second step, the microsphere was placed inside the cladding tube, according to Fig. 14. The fiber core was formed by injecting POMC prepolymer liquid into the cladding tube. To seamlessly integrate the cladding and core, thermal bonding was done at 70 °C for 3 days and 80 °C for 3 days.

**Figure 15:**
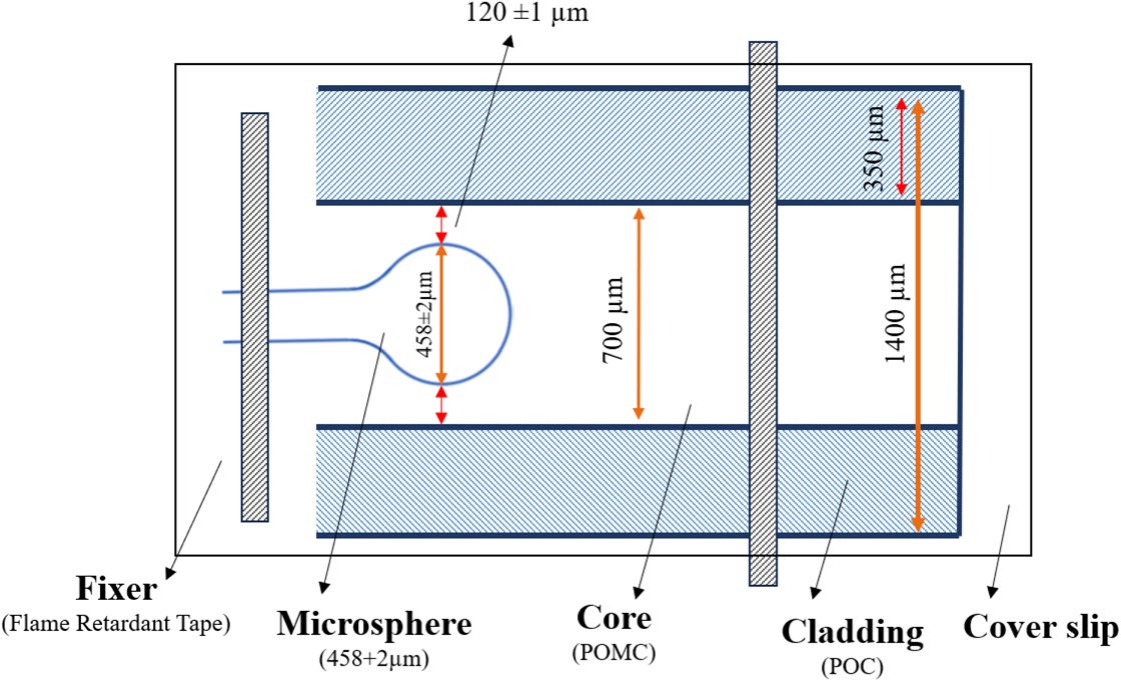
The schematic of fixing the microsphere inside the POC tube as cladding of POF.

During the fabrication process and thermal bonding, the shape and position of the microspheres remain unchanged. Fused silica fiber optic was used for microsphere fabrication, and thermal curing at 80 ºC did not affect the microsphere shape. To ensure the position of the microsphere in the POF, it was fixed using tape into the cladding tube, as shown in Fig. 15, and while filling the POC cladding with POMC, the position of the microsphere was controlled under the microscope. On the other hand, the high viscosity of the core material prevents the microspheres from moving inside the cladding^41,42^.

**Figure 16:**
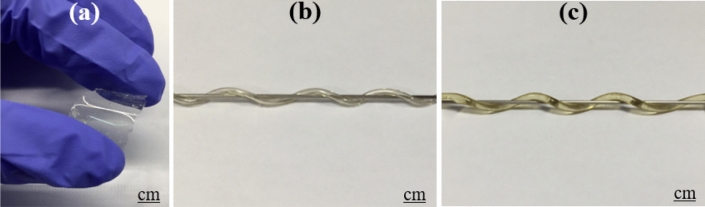
(**a**) Flexibility and mechanical properties of polymer POMC, and (**b**) POC. (**c**) A citrate-based POF twisted around a tube.

Ultimately, a stepped index polymer fiber 3–7 cm long with a diameter of 1400 µm was obtained. As shown in Fig. 16, POC and POMC polymers were flexible and had the same mechanical properties. The optical fiber can be easily bent around the rod.

**Figure 17:**
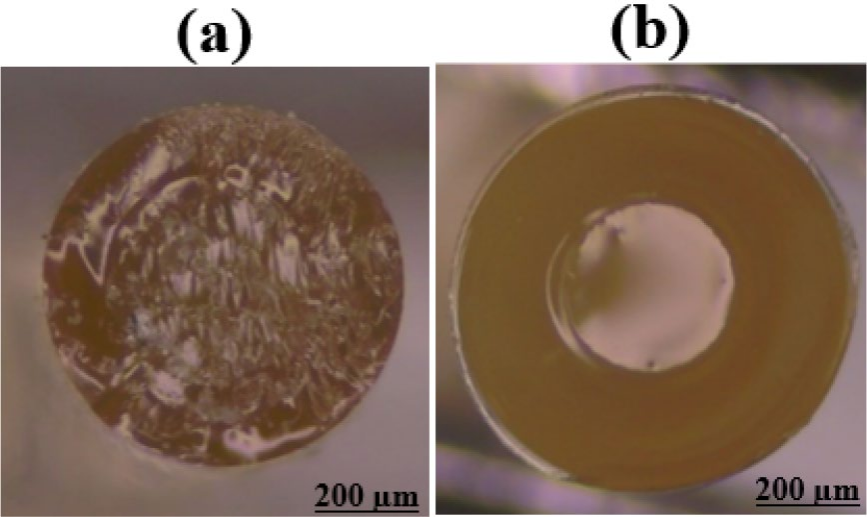
A photo of the POF tip (**a**) Cutting using a surgical blade. (**b**) After chemical etching.

For cleaving the tip of POF, a precise chemical etching method has been used to reduce surface roughness that leads to optical loss. The process involves cutting the tip of the POF using a surgical blade, followed by immersion in a 1 M NaOH solution for 60 s and three washes in water. This method ensures a smooth and precise cleaving of the POF. Figure 17 shows the tip of the POF before and after cleaving.

**Figure 18:**
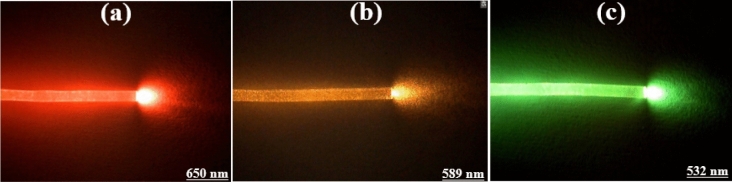
Guiding light in homemade POF at the wavelengths of (**a**) 650 nm, (**b**) 589 nm, and (**c**) 532 nm.

The light was coupled at three wavelengths 532 nm, 589 nm, and 650 nm as commercial wavelengths in biophotonics and neurophotonics applications to measure light coupling efficiency using a microsphere to the POF. As shown in Fig. 18, the designed flexible POF has the properties of a waveguide. Therefore, the light transmission efficiency of the POF has been obtained to be 77.8%, enhanced ~ 1.3 times rather than the previous report^11^.

#### Immobilizing the AuNPs on POF

**Figure 19:**
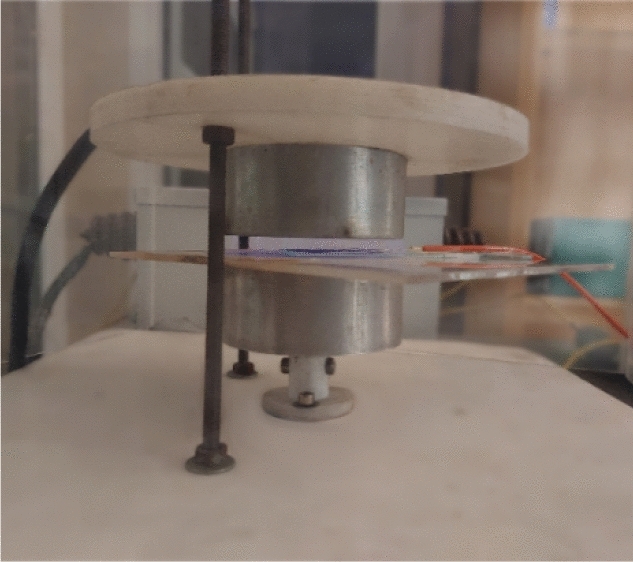
Fiber optics in a plasma device.

**Figure 20:**
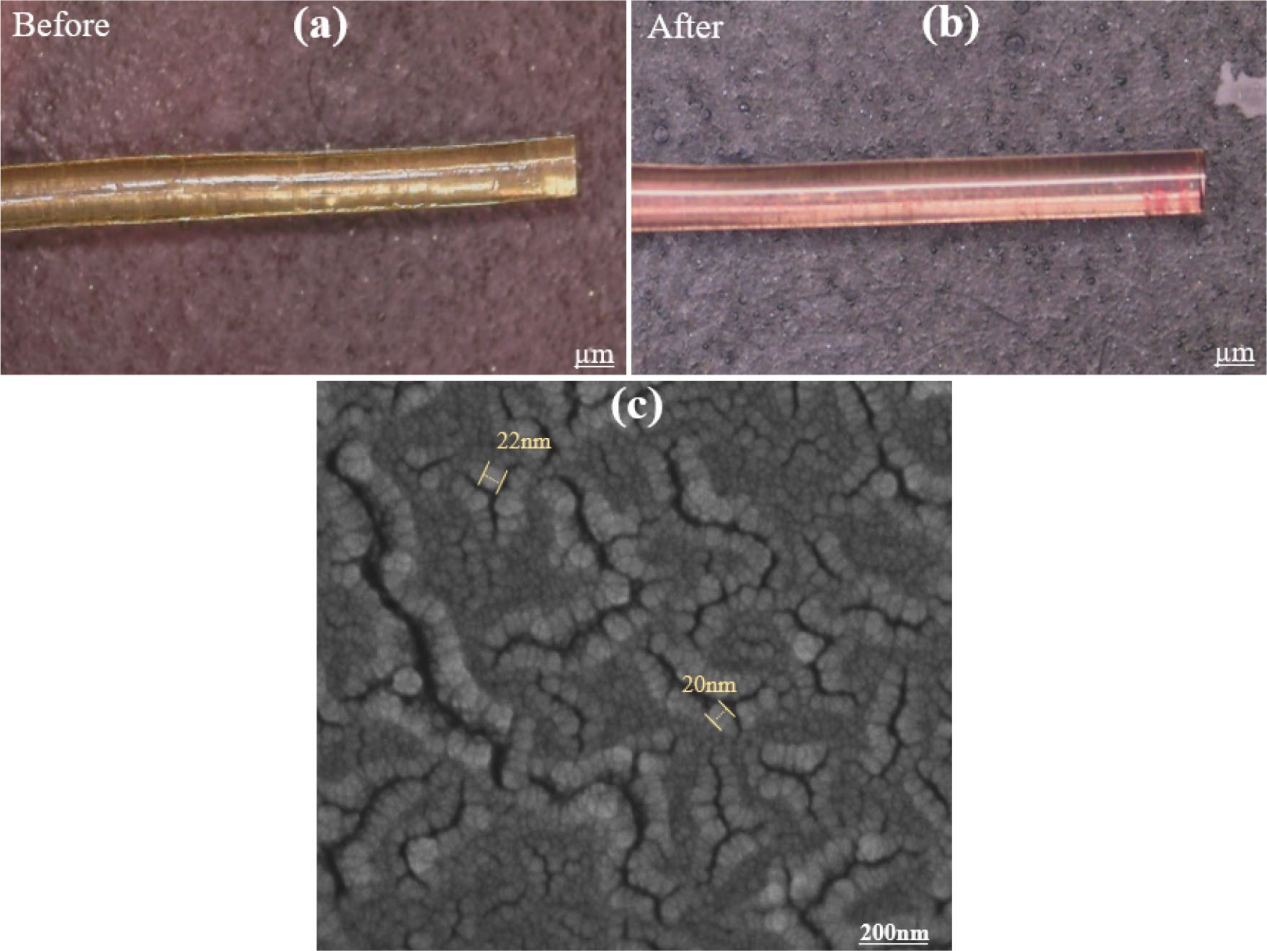
(**a**)The POF before, and (**b**) the POF after AuNPs immobilization. (**c**) FE-SEM image of NPs immobilized on the POFsurface.

The polymer used in making fiber optics was hydrophobic and limited the adhesion of AuNPs on its surface. Atmospheric plasma treatment was used to activate the POF surface and immobilize the AuNPs on the POF surface. The plasma treatment setup is shown in Fig. 19. The POF was exposed to the plasma for 5 min. After activating the surface of the POF, a length of 2 cm was immediately immersed in 3 mL of synthesized AuNPs solution. The immobilization process was carried out at room temperature for 14 h. It was then removed from the solution and allowed to dry at room temperature. Figure 20 shows photos of the before and after immobilizing AuNPs on the POF. As can be seen, the color of the POF surface has been changed from transparent to bright red. Besides, Fig. 20 shows the FE-SEM image of the immobilized POF surface, indicating that AuNPs have been successfully immobilized on the POF surface.

#### RI measurement using LSPR-POF

**Figure 21:**
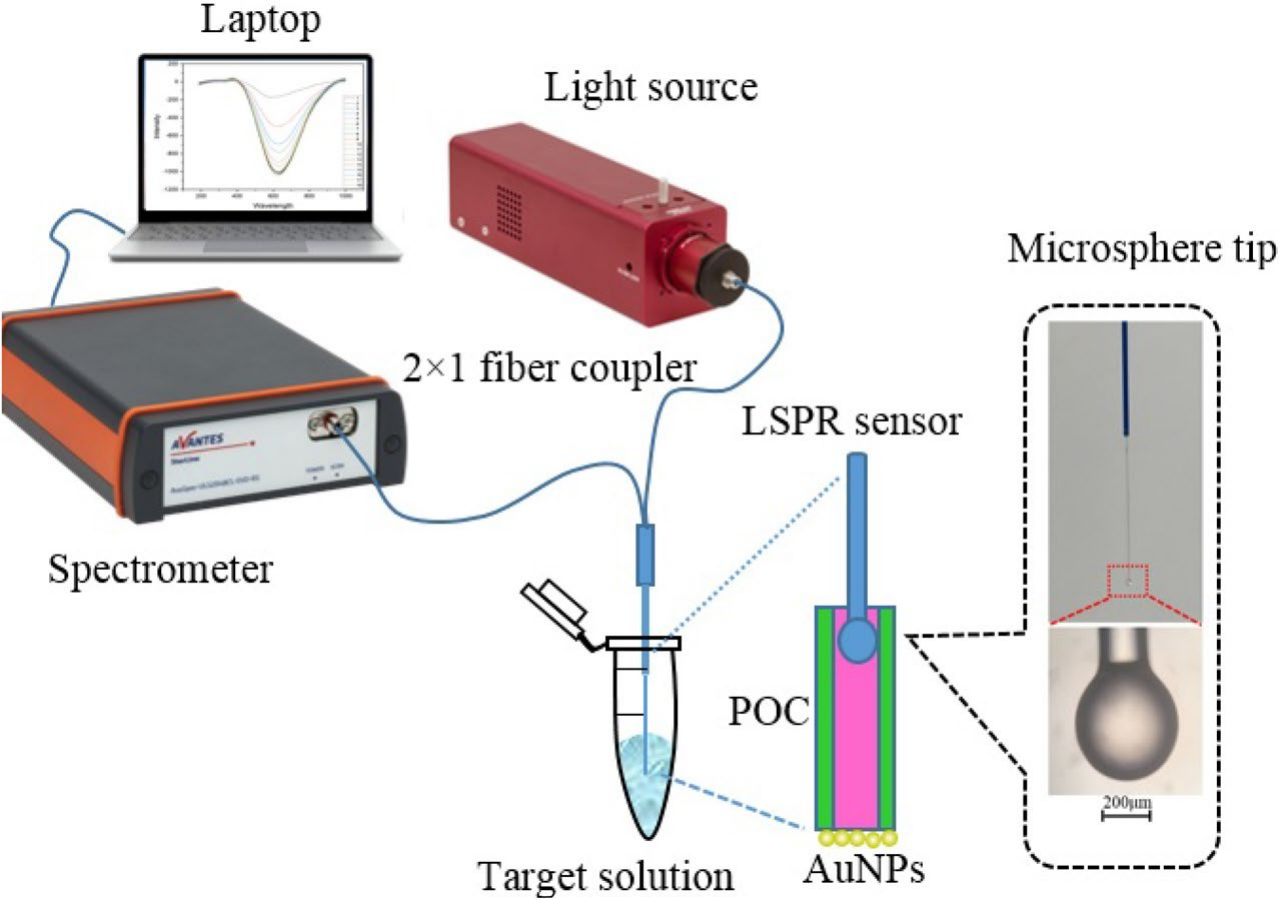
Schematic of the experimental setup.

The transmission spectrum was measured at different times to control the immobilization of the NPs. The experimental setup is shown in Fig. 21. Light from a stabilized tungsten-halogen light source (Thorlabs, SLS201L/M) passes through a 2 × 1 coupler and arrives at the sensor, where the reflected signal is detected using a spectrometer (Avantes, Avaspec-3648). The reflected spectrum was recorded for 800 min to monitor AuNPs immobilization.

Notably, the temperature can affect the sensor behavior in two different ways. The temperature may induce alterations in the polymer's structure or impact the NPs’ structure, leading to a shift in the oscillation frequency^43,44^. As per the findings and Fig. 3, the polymer's configuration stays consistent and unaltered at temperatures exceeding 30 ℃. Additionally, the optical fiber was produced at a temperature of 80 ℃; thus, exposing it to this temperature will not result in any structural changes.

Temperature above 50 ºC affects Au NP morphology and optical properties, leading to changes in the LSPR properties^43,44^. However, all the experiments were conducted at room temperature to avoid temperature effects.

**Figure 22:**
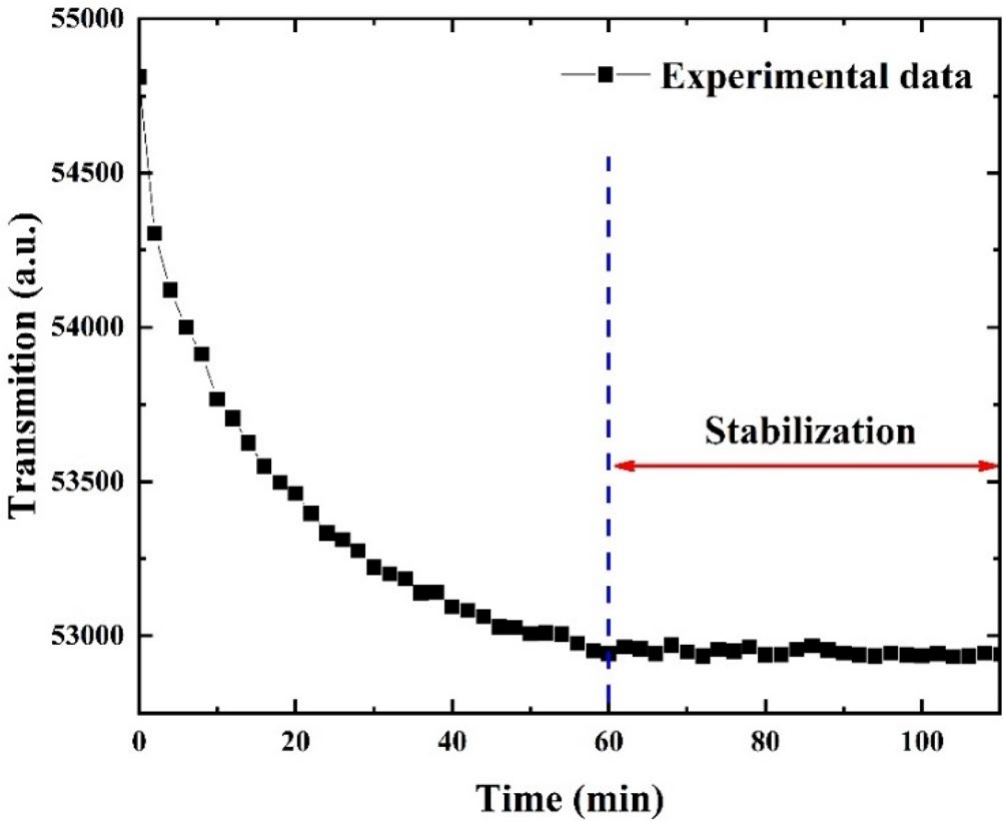
Stability of light source after 60 min warming up.

**Figure 23:**
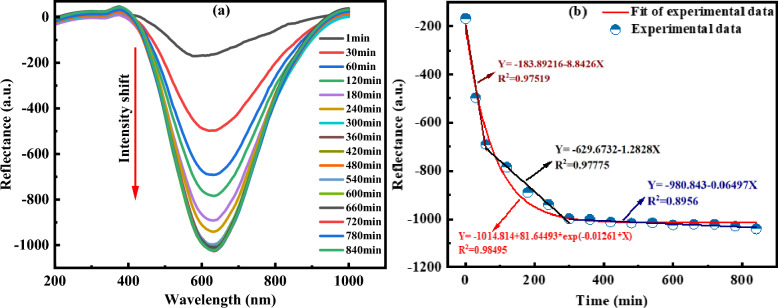
(**a**) Reflectance of the LSPR spectrum. (**b**) Changing the reflectance versus time during AuNPs immobilization.

Figure 22 shows the light source stability which after 60 min reached about 0.05% and then remained stable. As shown in Fig. 23a, during the immobilization of AuNPs on the tip of POF, transmissions decreased over time. Figure 23b shows the change in LSPR amplitude versus time, and the LSPR wavelength was obtained at 625 nm. The obtained results showed that the immobilization rates of NPs on the POF during 0 to 60 min, 60 to 300 min, and 300 to 840 min were -8.843 AU/min, -1.283 AU/min, and -0.06497 AU/min, respectively. Therefore, after 300 min, the output spectrum of the sensor was stable, and NP immobilization was completed.

Adsorption kinetics is an essential factor that determines the effectiveness of sorption^45^. Two models, the pseudo-first-order and pseudo-second-order models, are typically used to predict adsorption kinetics. The pseudo-first-order model assumes that the rate-limiting step is the physical adsorption process based on van der Waals or hydrogen bond forces. In contrast, the pseudo-second-order model assumes that the rate-limiting step is the chemisorption of the adsorbate onto active adsorption sites. The latter involves electron sharing or exchange between the adsorbent and adsorbate. The linear form of the pseudo-first-order model can be described as follows^46^:

7 $$\text{ln}\left({q}_{e}-{q}_{t}\right)=\text{ln}({q}_{e})-kt$$

where *k* is the pseudo-first-order rate constant (min^−1^), and *q*_*t*_ and *q*_*e*_ are the reflectance amounts at time *t* and equilibrium, respectively.

**Figure 24:**
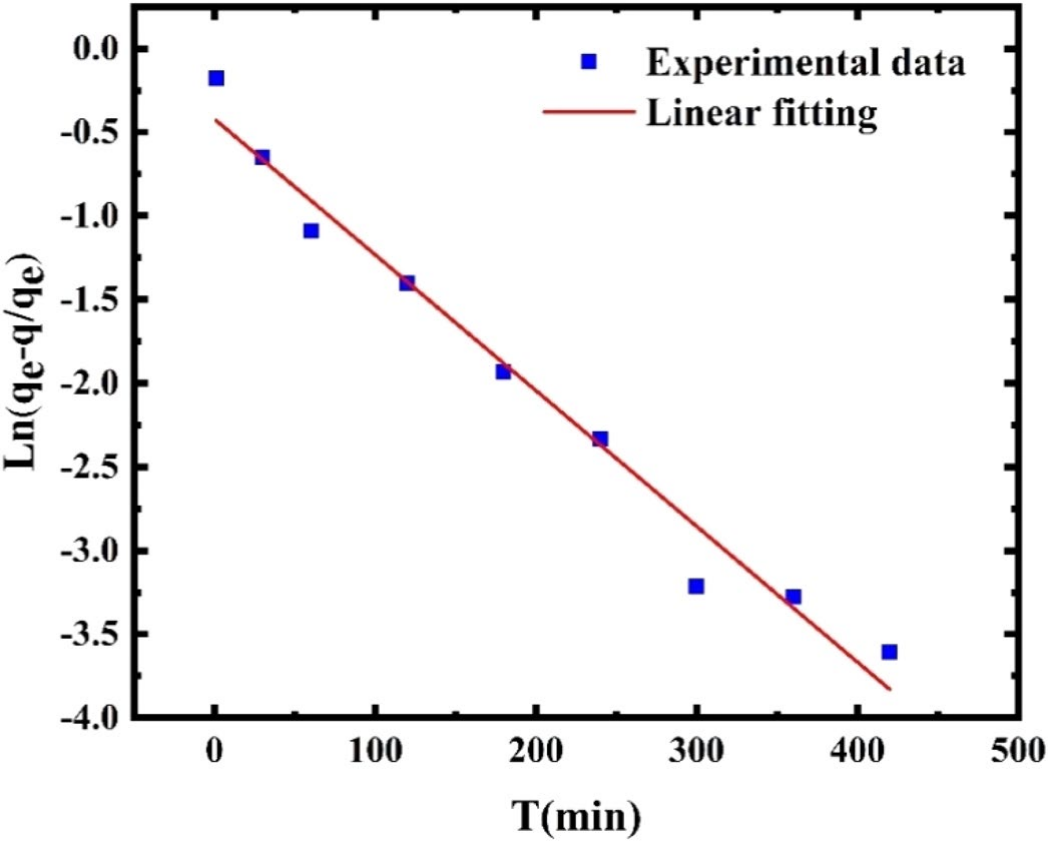
Pseudo-first-order kinetic model for the adsorption of AuNPs onto POF.

During adsorption, the reflectance − time curve is shown in Fig. 24. The adsorption rate gradually decreased until it reached equilibrium. At that point, no significant increase was found in the adsorption of AuNPs onto POF. Based on the adsorption kinetic curve, the adsorption rate constant (k) was determined to be 8.6 × 10^–3^ min^-1^. The correlation coefficient obtained from the pseudo-first-order kinetic model was 0.98, indicating a good fit with the experimental data.

**Figure 25:**
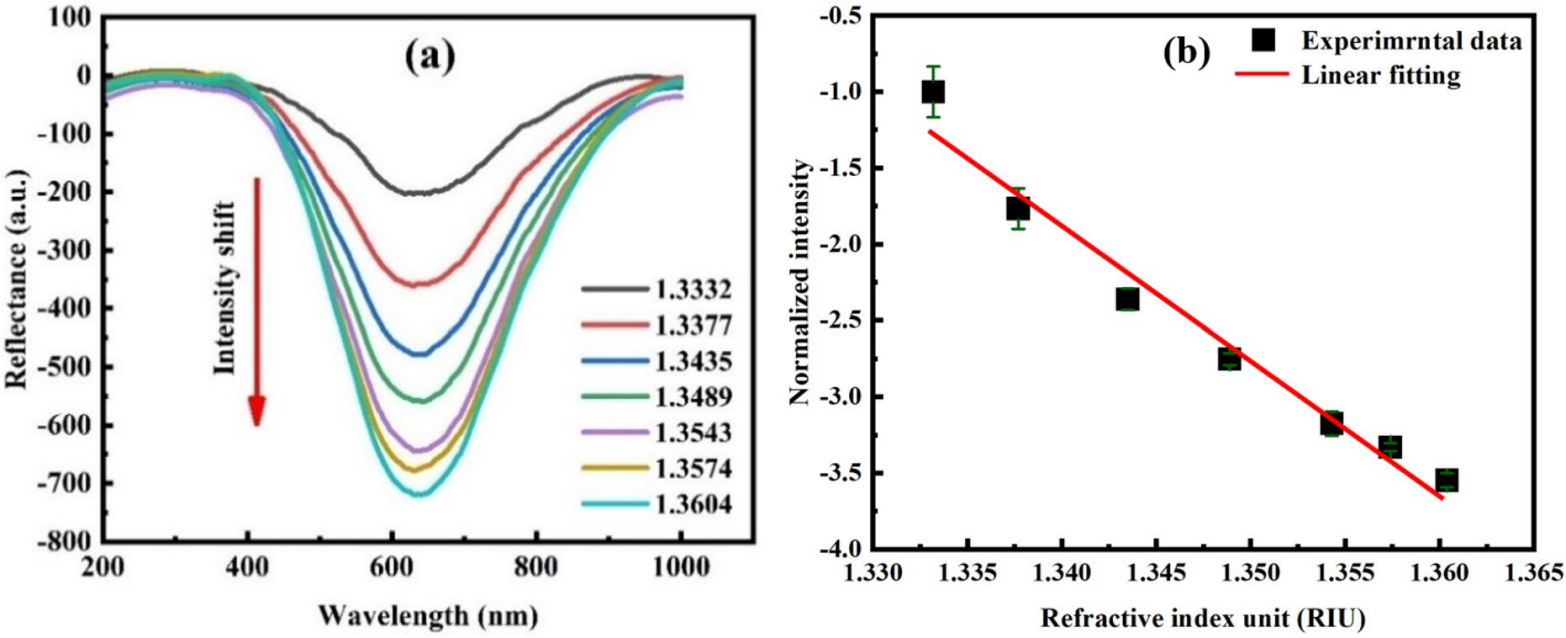
(**a**) Normalized LSPR spectrum for different ethanol concentrations. (**b**) Power intensity changes against RI with a 0.16 AU error bar.

The sensor was immersed in the different RIs in the range of 1.3332 to 1.3604 RIU to measure the RI sensitivity within the range of RI of biological samples^47,48^. To achieve this purpose, ethanol solutions with 0% -60% (V/V) concentrations were used. As shown in Fig. 25a, with the increase in ethanol concentration, the corresponding resonance peak changed from − 202.13 AU to − 719.15 AU. The intensity sensitivity of the POF sensor is given by:

8 $$\text{S} = \frac{\Delta I/{I}_{0}}{\Delta n}\times 100(\text{\%}/RIU)$$

The linearity of intensity changes versus RI for the three-time experiment is shown in Fig. 25b, where the RI sensitivity of 7778%/RIU was observed for the AuNPs-decorated POF with R^2^ = 0.99 and 0.16 AU error bar, representing an increase by a factor of ~ 5 compared to the narrow groove POF coated with an Au film^49^. In addition, the experimental and modelling results were in incredible agreement.

**Figure 26:**
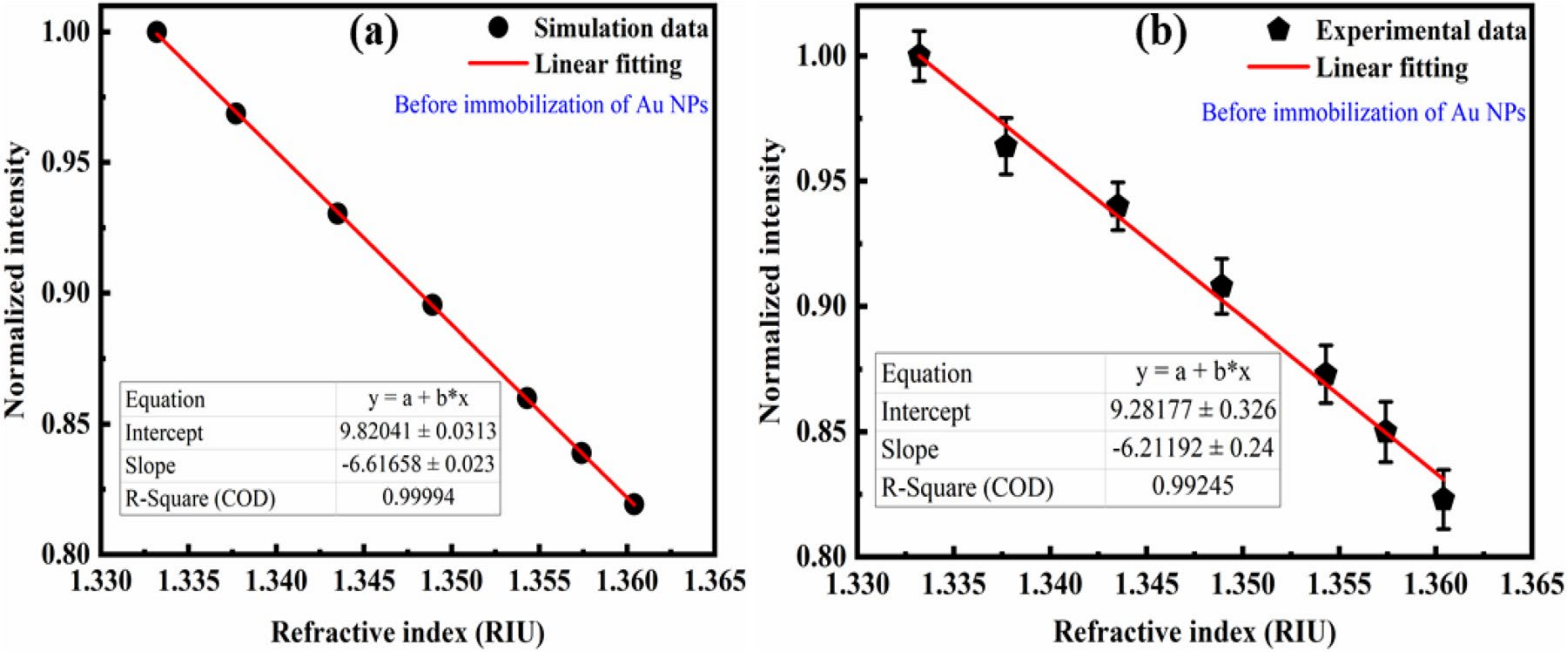
RI sensitivity of the POF tip before Au NPs immobilization obtained from (**a**) FEM simulation, and (**b**) experimental results based on intensity modulation.

Furthermore, the RI sensitivity of the POF tip before Au NPs immobilization in the range of 1.3332–1.3604 RIU was evaluated experimentally and FEM simulation. Figure 26 shows that the experimental result was in good agreement with the simulation result, and the RI sensitivity of the POF tip in terms of FEM simulation and experiment is 661.66%/RIU and 621.2%/RIU, respectively. Notably, the maximum measurement errors are approximately 1.1%. This study presents the design and development of an LSPR-POF sensor that can enhance the RI sensitivity about 12 times compared to the before Au NPs immobilization on the POF tip.

The obtained results show that the designed sensor can be used as an excellent sensor for biosensor applications. To the best of our knowledge, this report marks the first instance of a flexible and degradable fiber optic LSPR based on POF. Table 2 outlines a comparison of RI sensor sensitivities reported in the literature, utilizing different techniques for measurement through intensity modulation. After analyzing the results presented in Table 2, several useful conclusions can be drawn. Intensity modulation POF-based RI sensors can measure the RI, ranging from 1.333 (water) to an RI close to that of the fiber core/cladding. Demonstratively, the RI sensitivities differ among POFs with various structures, indicating that this sensor type is suitable for general RI measurement applications. Additionally, the LSPR-POF structure demonstrates higher sensitivity. The suggested POF sensor has the potential to detect liquid RI through intensity modulation, offering advantages such as simplicity, ease of implementation, biodegradability, and cost-effectiveness.

**Table 2:** Comparison of various RI fiber optic sensors reported in the literatures based on intensity modulation.

Sensor structure	RIU	Sensitivity	Year	Ref
Optical fiber Michelson interferometer	1.30–1.43	− 202.46 dB/RIU	2015	^50^
No-core fiber filter (NCFF)	1.3329–1.3781 1.3781–1.4010	− 99.191 dB/RIU − 139.958 dB/RIU	2017	^51^
U-shaped multimode fiber sensor	1.3405–1.3874 1.3874–1.4223	− 115.09 dB/RIU − 260.24 dB/RIU	2018	^52^
Tip fiber intermodal interferometer	1.3373–1.3904	112.2dB/RIU	2018	^53^
LSPR tapered fiber optic	1.328–1.379	2032%/RIU	2023	^54^
Slanted end fiber optic	1.3095–1.3439	− 136 dBm/RIU	2023	^55^
Narrow groove plastic optical fiber coated with Gold film	1.340–1.356	1432%/RIU	2020	^49^
U-shaped plastic optical fiber	1.33–1.41	− 1121.4%/RIU	2022	^56^
SPR photonic crystal fiber	1.32–1.39	328%/RIU	2023	^57^
SPR D-shape fiber sensor	1.334–1.346	3.101 au/RIU	2023	^58^
Long period grating flat-shaped plastic optical fiber	1.3330–1.4230	6563%/RIU	2023	^59^
Parylene-mediated plasmonic–photonic cavity	1.333–1.397	3.148%/RIU	2023	^60^
Plastic optical fiber balloon structure	1.3403–1.3579	3105%/RIU	2022	^61^
Tip nanodisk plasmonic fiber optic	1.33–1.38	9.61 dB/RIU	2024	^62^
Tip LSPR fiber optic	1.33–1.38	7.08 dB/RIU	2024	^63^
Tapered LSPR fiber optic	1.3324–1.4254	0.967 au/RIU	2020	^64^
MXene SPR fiber optic	1.3343–1.3765	57.2%/RIU	2020	^65^
Double-sided polished U-shaped plastic optical fiber	1.33–1.39	1541%/RIU	2020	^66^
Long period grating plastic optical fiber	1.33–1.45	2815%/RIU	2019	^67^
Side-polished macrobend plastic optical fiber	1.33–1.41	43 dB/RIU	2019	^68^
Screw-shaped plastic optical fiber	1.37–1.40 1.41–1.45	4318%/RIU 4399%/RIU	2020	^69^
Twisted tapered plastic optical fibers	1.37–1.41	1700%/RIU	2019	^70^
Side-polished U-shaped plastic optical fiber	1.33–1.44	864%/RIU	2017	^71^
D-shaped plastic optical fiber	1.333–1.41	800%/RIU	2014	^72^
U-shaped, multi-notched plastic optical fiber	1.333–1.410	1130%/RIU	2017	^73^
Tip LSPR POF	1.333–1.360	7767%/RIU		This paper

## Conclusion

Citrate-based polymer fiber optic probes are made of two polymers, poly (octamethylene citrate) (POC) and poly (octamethylene maleate) (POMC). These probes are highly flexible and biodegradable in biological tissues, making them ideal for creating an LSPR sensor. The biodegradability of these probes in body tissues addresses a critical issue in medical science, which is tissue damage. A polymer optical fiber with a length of 7 cm and a diameter of 1400 µm, with suitable light transmission and an efficiency of 77.8% can be used as a tool for an LSPR sensor. To make an RI sensor, AuNPs with a size of 20 nm were immobilized on the POF using the plasma method. The POF tip surface becomes saturated with AuNPs immobilization in 840 min, and the maximum rate of NP immobilization was obtained to be -8.843 AU/min in 60 min. The adsorption kinetics of AuNPs immobilization on the POF, which is responsible for sorption efficiency, follows the pseudo-first-order model. From the adsorption kinetic curve, the adsorption rate constant was 8.6 × 10^–3^ min^−1^. The correlation coefficient obtained from the pseudo-first-order kinetic model is 0.98. Finally, the RI sensitivity in the range of 1.3332 to 1.3604 RIU was obtained to be 7778% RIU^−1^ for the AuNPs-decorated POF, representing an increase by a factor of ~ 5 compared to the narrow groove POF coated with Au film^37^. Moreover, the experimental results demonstrated that the proposed sensor exhibits favorable output response characteristics consistent with the simulation results.

The present results show that this sensor can be used for biosensing applications. Additional bio-functional modifications must be made to the fiber to measure the changes in the RI of a specific substance. This has potential application value in biochemical sensing, particularly in using biodegradable fiber optic biosensors to sense neurotransmitters in neuroscience applications. The proposed structure is sensitive, biocompatible, non-destructive, and immune to electromagnetic interference, making it well-suited for monitoring action potentials and neural activity.

## Data Availability

All data analyzed during the current study are available from the corresponding author on reasonable request.
